# Phosphorylated neurofilament heavy chain in cerebrospinal fluid and plasma as a Nusinersen treatment response marker in childhood-onset SMA individuals from Serbia

**DOI:** 10.3389/fneur.2024.1394001

**Published:** 2024-05-02

**Authors:** Miloš Brkušanin, Ana Kosać, Vesna Branković-Srećković, Kristina Jovanović, Stojan Perić, Jelena Karanović, Suzana Matijašević Joković, Nemanja Garai, Jovan Pešović, Dimitrije Nikolić, Zorica Stević, Goran Brajušković, Vedrana Milić-Rašić, Dušanka Savić-Pavićević

**Affiliations:** ^1^Faculty of Biology, Centre for Human Molecular Genetics, University of Belgrade, Belgrade, Serbia; ^2^Clinic for Neurology and Psychiatry for Children and Youth, Belgrade, Serbia; ^3^University Children's Hospital Tirsova, University Clinical Centre of Serbia, Belgrade, Serbia; ^4^Neurology Clinic, University Clinical Centre of Serbia, Belgrade, Serbia; ^5^School of Medicine, University of Belgrade, Belgrade, Serbia

**Keywords:** spinal muscular atrophy, phosphorylated neurofilament heavy chain (pNFH), Nusinersen, biomarker, antisense oligonucleotide, cerebrospinal fluid

## Abstract

**Introduction:**

Biomarkers capable of reflecting disease onset and short- and long-term therapeutic effects in individuals with spinal muscular atrophy (SMA) are still an unmet need and phosphorylated neurofilament heavy chain (pNF-H) holds significant promise.

**Methods:**

We conducted a longitudinal prospective study to evaluate pNF-H levels in the cerebrospinal fluid (CSF) and plasma of 29 individuals with childhood-onset SMA treated with Nuinersen (SMA type 1: *n* = 6, 2: *n* = 17, 3: *n* = 6). pNF-H levels before and during treatment were compared with the levels of controls (*n* = 22), patients with Duchenne muscular dystrophy (*n* = 17), myotonic dystrophy type 1 (*n* = 11), untreated SMA individuals with chronic type 3 disease (*n* = 8), and children with presymptomatic SMA (*n* = 3).

**Results:**

SMA type 1 showed the highest mean CSF pNF-H levels before treatment initiation. All Nusinersen-treated individuals (types 1, 2, and 3) showed significantly elevated mean baseline CSF pNF-H compared to controls, which inversely correlated with age at disease onset, age at first dose, disease duration and the initial CHOP INTEND result (SMA type 1 and 2). During 22 months of treatment, CSF pNF-H levels declined during loading doses, stabilizing at reduced levels from the initial maintenance dose in all individuals. Baseline plasma pNF-H levels in type 1 and 2 SMA were significantly increased compared to other cohorts and decreased notably in type 1 after 2 months of treatment and type 2 after 14 months. Conversely, SMA type 3, characterized by lower baseline pNF-H levels, did not show significant fluctuations in plasma pNF-H levels after 14 months of treatment.

**Conclusion:**

Our findings suggest that CSF pNF-H levels in untreated SMA individuals are significantly higher than in controls and that monitoring of CSF pNF-H levels may serve as an indicator of rapid short-term treatment response in childhood-onset SMA individuals, irrespective of the subtype of the disease, while also suggesting its potential for assessing long-term suppression of neurodegeneration. Plasma pNF-H may serve as an appropriate outcome measure for disease progression and/or response to treatment in types 1 and 2 but not in type 3. Presymptomatic infants with SMA may show elevated pNF-H levels, confirming early neuronal degeneration.

## 1 Introduction

Spinal muscular atrophy (SMA) was the primary genetic cause of infant mortality in the era before innovative genetically designed therapies (1). It is characterized by the degeneration of alpha motor neurons in the anterior horns of the spinal cord, due to the lack of the SMN (survival of motor neurons) protein. Prior to the approval of genetically designed therapies, a broad and continuous spectrum of childhood-onset SMA phenotypes was categorized into different clinical types (1, 2 and 3) based on the age of symptom onset and achieved motor milestones in SMA patients (2), with SMA type 1 being the most severe form of the disease at one end of the spectrum and SMA type 3 as the mildest form at the other end. Despite variations in clinical severity, all individuals affected by SMA share a common genetic cause. Approximately 95–98% of SMA cases are attributable to the homozygous absence of the *SMN1* gene (OMIM# 600354), which results in a scarcity of the SMN protein (3). About 2%–5% of cases are compound heterozygotes, with the absence of one *SMN1* copy and a small mutation (point mutation, small insertion, small deletion) in the remaining copy (4). The presence of the paralogous *SMN2* gene (OMIM# 601627) prevents embryonic lethality which would be caused by the complete absence of the SMN protein. However, the *SMN2* gene primarily produces a transcript lacking exon 7, resulting in the production of only ~10% of the functional SMN protein (3). The number of copies of the *SMN2* gene can vary among individuals and typically, a higher number of *SMN2* copies in an SMA individual correlates with a milder form of the disease (5, 6). Nonetheless, it is important to note that this correlation is not always definitive.

There has been significant progress in recent years in the development of treatments for SMA. Antisense oligonucleotide, Nusinersen, has been the first genetically designed therapy approved for the treatment of all types of SMA. It functions by correcting the splicing defect in *SMN2*, facilitating the inclusion of exon 7 and thereby increasing the production of the functional SMN protein from each *SMN2* copy (7). The administration of Nusinersen in all SMA individuals involves an initial series of four loading doses during the first 2 months, followed by regular lifelong maintenance doses administered every 4 months (8). Since antisense oligonucleotides do not pass the blood–brain barrier, the therapy is delivered directly into the spinal canal through a procedure that begins with the collection of cerebrospinal fluid (CSF) samples, which is a fundamental requirement for the therapeutic injection. Currently, the only clinical indicators of better response to treatment are a later age at disease onset and higher scores of motor function (9). Due to the increasing number of SMA individuals treated with Nusinersen, there is a growing need for sensitive, reliable, and objective biomarkers that can reflect short- and long-term therapeutic effects and potentially detect changes before they manifest in motor performance.

Neurofilament proteins, which are specific to neurons, can be detected in the CSF and plasma upon neuronal degeneration. Their levels have consistently been associated with axonal degeneration in various neurodegenerative diseases (10). Recent research suggests that levels of phosphorylated neurofilament heavy chain (pNF-H) could serve as a response biomarker in SMA individuals undergoing Nusinersen treatment (11–13).

In order to gain a more comprehensive understanding of a potential role of pNF-H as a biomarker for disease activity and treatment response during Nusinersen therapy, we performed a longitudinal examination of CSF and plasma levels of pNF-H in individuals diagnosed with SMA types 1, 2, and 3 and treated with Nusinersen. In addition, to investigate the potential disease- and motor neuron-specific nature of pNF-H levels, our study included samples from 50 non-SMA individuals: control individuals without inherited motor-neuron and neuromuscular disorders and individuals suffering from Duchenne muscular dystrophy (DMD) and myotonic dystrophy type 1 (DM1).

## 2 Materials and methods

This study was conducted in accordance with the principles outlined in the Declaration of Helsinki. After being informed about the study design and aims, all participants and the parents of minors provided written informed consent for the collection, storage, and analysis of samples in accordance with the regulations of the local ethics committees.

### 2.1 Individuals with SMA

This prospective longitudinal cohort study enrolled 29 genetically diagnosed, childhood-onset and Nusinersen treated SMA individuals from the time of the drug approval in Serbia, spanning the period from July 2018 to January 2023 (Table 1). The *SMN2* copy number was determined in all SMA individuals using multiplex ligation-dependent probe amplification (MLPA). For SMA type 1 (n = 6; 3 males, 3 females), recruitment took place at the University Children’s Hospital Tirsova in Belgrade (UCHT). Among them, one individual was followed for 2 years, while five individuals were lost to follow-up: three received six doses of Nusinersen (two switched to gene therapy, and one passed away) and two received four doses of Nusinersen before switching to gene therapy. SMA type 2 individuals (*n* = 17; 9 males, 8 females) were recruited at the Clinic for Neurology and Psychiatry for Children and Youth in Belgrade (CNPCY) and received nine doses of Nusinersen treatment. SMA type 3 group (*n* = 6) consisted of two individuals with type 3a (1 male, 1 female) recruited from CNPCY, and four individuals with type 3b (4 females) recruited from the Neurology Clinic at the University Clinical Centre of Serbia in Belgrade (NCUCCS). All received nine doses of Nusinersen.

**Table 1:** Demographics, genetics and treatment characteristics of Nusinersen treated SMA subjects.

Subject	SMA type	Gender	*SMN2* copy number	*SMN1* copy number	Number of received Nusinersen doses
SMA1	1	M	2	0	6
SMA2	1	F	2	0	9
SMA3	1	F	2	0	6
SMA4	1	F	2	0	6
SMA5	1	M	2	0	4
SMA6	1	M	2	0	4
SMA7	2	F	3	0	9
SMA8	2	F	3	0	9
SMA9	2	M	3	0	9
SMA10	2	F	3	0	9
SMA11	2	F	3	0	9
SMA12	2	M	3	0	9
SMA13	2	M	3	0	9
SMA14	2	F	3	0	9
SMA15	2	M	3	0	9
SMA16	2	M	3	0	9
SMA17	2	F	3	0	9
SMA18	2	M	3	0	9
SMA19	2	F	3	0	9
SMA20	2	F	3	0	9
SMA21	2	M	3	0	9
SMA22	2	M	3	0	9
SMA23	2	M	3	0	9
SMA24	3a	F	3	0	9
SMA25	3a	M	2	1	9
SMA26	3b	F	3	0	9
SMA27	3b	F	3	0	9
SMA28	3b	F	2	1	9
SMA29	3b	F	4	0	9
SMA30	Presymptomatic	M	4	0	6

SMA, spinal muscular atrophy; M, male; F, female. Individuals with SMA possessing one copy of the SMN1 gene were compound heterozygotes carrying the c.821C>T variant within exon 6. Two presymptomatic individuals with SMA, identified by newborn screening for the condition, were treated with Risdiplam and were not included in the table.

The study also included three clinically silent SMA individuals, referred to as SMA NBS (1 female and 2 males), identified through the *Feasibility study of newborn screening for SMA in Serbia*. Among them were two newborns from unrelated families, treated with Risdiplam, as well as an older sibling of one newborn who received Nusinersen. Additionally, eight treatment-naïve SMA individuals (1 male, 7 females) were recruited at the CNPCY.

Demographic and clinical data were collected from all individuals. Children diagnosed with SMA types 1 and 2 under 2 years of age underwent assessment utilizing the Children’s Hospital of Philadelphia Infant Test of Neuromuscular Disorders (CHOP-INTEND; maximum score is 64 points) and for those older than 2 years with SMA type 2, the assessment included the Hammersmith Infant Neurological Examination Section 2 (HINE-2) scale (maximum score is 56 points). Individuals with SMA types 2 and 3 underwent evaluation using the Hammersmith Functional Motor Scale Expanded (HFMSE; maximum score is 66 points). The Revised Amyotrophic Lateral Sclerosis Functional Rating Scale (ALSFRS-R) was utilized to assess motor function in individuals with type 3b SMA (maximum score is 48 points). Motor function evaluations were carried out at baseline, as well as at 12 months and 24 months following the initiation of treatment.

CSF samples were obtained immediately prior to each intrathecal administration of Nusinersen: at baseline (visit 1), day 15 (visit 2), day 29 (visit 3), day 64 (visit 4), day 183 (visit 5), day 302 (visit 6), day 422 (visit 7), day 540 (visit 8), and day 659 (visit 9). Following collection, the CSF samples were divided into smaller aliquots and immediately stored at a temperature of −80°C.

From all subjects with SMA treated with Nusinersen, plasma samples were collected on two separate occasions: initially at baseline and subsequently after a 2-month interval for SMA type 1 (before the 4th dose) or a 14-month interval for types 2 and 3 (before the 7th dose). The second blood sampling for treatment-naïve SMA subjects occurred after a period of 3 years and among the initial eight subjects, only four were available in the second collection. SMA NBS individuals were sampled once at baseline. Blood samples were collected in K2E EDTA tubes and centrifuged at 2,000 ×g for 15 min at a temperature of +4°C, the plasma component was separated, divided into cryovials, and promptly frozen at a temperature of −80°C within a maximum of 1 h after sampling.

### 2.2 Individuals without SMA

The cohort consisted of individuals with genetically confirmed DMD (*n* = 17; all males; median age at sampling was 8 years [interquartile range (IQR): 8–9]) and DM1 (*n* = 11; 6 males, 5 females; median age at sampling was 31 years [IQR: 27.2–32.5]), as well as individuals without inherited motor-neuron and neuromuscular disorders (*n* = 22; 9 males, 13 females; median age at sampling was 9.1 years [IQR: 5–13]), hereafter referred to as controls. Individuals with DMD were recruited from UCHT, while individuals with DM1 were recruited from NCUCCS. Control individuals were recruited from UCHT and CNPCY. Peripheral blood samples were collected only once from all participants, and plasma was separated following the aforementioned protocol. Additionally, for control individuals (n = 9), a lumbar puncture was performed as part of diagnostic procedures under suspicion of various diseases (primary headaches, benign intracranial hypertension, and functional neurological disorders), and the CSF samples were used for pNF-H level analysis.

### 2.3 Sample analysis

To measure the pNF-H levels in CSF and plasma, a commercially available Phosphorylated Neurofilament H Human ELISA assay (BioVendor R&D, Czech Republic) was used following the manufacturer’s instructions. The absorbance measurement at wavelengths of 450 nm and 630 nm was performed using the Epoch™ Microplate Spectrophotometer (BioTek, United States), equipped with Gen5 Microplate Reader and Imaging Software version 3.10 (BioTek, United States).

### 2.4 Statistical analysis

Descriptive statistics were used to analyze the demographic characteristics. Categorical variables were presented as numbers, age was presented as median [IQR], while other continuous variables were reported as mean ± standard deviation (SD).

To construct a standard curve for the quantification of pNF-H in the analyzed samples, GraphPad Prism version 8.2.1 for Windows (GraphPad Software, United States) was utilized. The standard curve was generated by plotting the mean absorbance of standards against the known concentrations of the standards using a logarithmic scale and the four-parameter algorithm. To facilitate statistical analysis, concentrations below the lower limit of quantification (BLQ) were assigned a value equivalent to half of the limit of detection, specifically 11.75 pg./mL.

The normality of data distribution within groups was evaluated using the Shapiro–Wilk test. Equality of variances was examined using the Levene’s Test. When the data deviated from normal distribution, the Kruskal-Wallis rank sum test was employed for comparisons among more than two groups, including baseline scores of CHOP-INTEND, and baseline pNF-H levels in CSF and plasma. Post-hoc analysis was conducted using Dunn’s Multiple Comparison Test. The Mann–Whitney *U*-test was utilized to compare two groups when the data deviated from normal distribution, such as baseline HFMSE scores, and for comparison of CSF pNF-H levels between baseline and after 10 or 22 months. In case of unequal variances, Welch’s *t*-test was used.

To examine the correlation between baseline pNF-H levels in CSF and various clinical characteristics of the disease (age at onset, age at first dose, disease duration at baseline, baseline CHOP-INTEND score, baseline HINE-2 score, baseline HFMSE score, as well as motor scores after 1 and 2 years), the Spearman correlation test was employed. All analyses were conducted with a significance level set at 0.05. Statistical tests and figure creation were performed using the R environment for statistical computing and visualization, version 4.3.1.

## 3 Results

### 3.1 Demographic and clinical characteristics of individuals with SMA

Table 2 provides a summary of the demographic and clinical characteristics of different SMA cohorts.

**Table 2:** Summary of demographic and clinical data of different SMA cohorts.

	SMA type 1	SMA type 2	SMA type 3	SMA NBS	naive SMA
**Characteristic**					
Gender	*n* = 6	*n* = 17	*n* = 6	*n* = 3	*n* = 8
Female, *n (%)*	3 (50)	8 (47)	5 (83)	1 (33)	7 (87.5)
Male, *n (%)*	3 (50)	9 (53)	1 (17)	2 (67)	1 (12.5)
Age at onset	*n* = 6	*n* = 17	*n* = 6	NA	*n* = 8
Median (IQR)	2 (2) m	7 (6–9) m	2.7 (2.1–3) y	NA	1.6 (1–3.6) y
Age at treatment	*n* = 6	*n* = 17	*n* = 6	*n* = 3	NA
Median (IQR)	5.6 (5.1–6.7) m	2.8 (1.6–4.4) y	28.4 (17.4–32.1) y	9.9 (0.7–8.4) m	NA
Treatment delay	*n* = 6	*n* = 17	*n* = 5	*	/
Median (IQR)	3.1 (3–4.5) m	2 (0.9–3.7) y	24.1 (12.2–25.2) y	/	/
*SMN2* copy number	*n* = 6	*n* = 17	*n* = 6	*n* = 3	*n* = 8
*2*	6	0	2	0	0
*3*	0	17	3	1	7
*4*	0	0	1	2	1
Baseline CHOP-INTEND	*n* = 6	*n* = 8	/	*n* = 3	/
Mean ± SD	15 ± 7.2	33.6 ± 7	/	60.7 ± 5.8	/
One-year CHOP-INTEND	*n* = 2	*n* = 7	/	/	/
Mean ± SD	33.5 ± 0.7	43 ± 5.9	/	/	/
Two-year CHOP-INTEND	*n* = 1	/	/	/	/
	44	/	/	/	/
Baseline HINE-2	*n* = 1	*n* = 17	*n* = 1	/	/
Mean ± SD	3	9.8 ± 3.4	24	/	/
One-year HINE-2	/	*n* = 16	/	/	
Mean ± SD	/	14 ± 3.6	/	/	/
Two-year HINE-2	/	*n* = 4	/	/	/
Mean ± SD	/	15.2 ± 5.4	/	/	/
Baseline HFMSE	/	*n* = 9	*n* = 5	/	/
Mean ± SD	/	13.1 ± 7.7	19.6 ± 17.2	/	/
One-year HFMSE	/	*n* = 12	*n* = 5	/	/
Mean ± SD	/	19.1 ± 8.6	24.2 ± 20.8	/	/
Two-year HFMSE	/	*n* = 14	/	/	/
Mean ± SD	/	23.6 ± 8.4	/	/	/
Baseline ALSFRS-R	/	*/*	*n = 4*	/	/
Mean ± SD	/	/	30 ± 3.9	/	/
One-year ALSFRS-R	/	/	*n* = 4	/	/
Mean ± SD	/	/	32 ± 3.6	/	/
Two-year ALSFRS-R	/	/	*n* = 4	/	/
Mean ± SD	/	/	34.5 ± 5.5	/	/

Categorical variables are presented as numbers, age is presented as median [interquartile range, IQR], while other continuous variables are reported as mean ± standard deviation (SD). SMA, spinal muscular atrophy; SMA NBS, SMA individuals identified by newborn screening; n, number; m, months; y, years; /, data not available; CHOP-INTEND, Children’s Hospital of Philadelphia Infant Test of Neuromuscular Disorders; HINE-2, Hammersmith Infant Neurological Examination Section 2; HFMSE, Hammersmith Functional Motor Scale—Expanded; ALSFRS-R, Revised Amyotrophic Lateral Sclerosis Functional Rating Scale. ^*^Presymptomatic SMA individuals diagnosed by newborn screening for the condition received immediate treatment upon diagnosis: two newborns were treated with Risdiplam at the age of 17 days, whereas the older sibling of one of them was administered Nusinersen at the age of 16 months.

Type 1 SMA individuals received Nusinersen at the age of 5.6 months [IQR: 5.1–6.7] and all had 2 copies of the *SMN2* gene. Type 2 SMA individuals received Nusinersen at the age of 2.8 years [IQR: 1.6–4.4] and all had 3 copies of the *SMN2* gene. Among the type 3 SMA individuals, type 3a received Nusinersen at the age of 10.4 years [IQR: 6.6–12.2]; one had 3 copies of *SMN2*, and the other was a compound heterozygote with 2 copies of *SMN2* and a c.821C>T variant within exon 6 of the *SMN1* gene. The remaining four individuals classified as type 3b received therapy when they were 31.4 years old [IQR: 28.4–34.8], two had 3 copies of *SMN2*, one had 4 copies of *SMN2*, and one was a compound heterozygote with 2 copies of *SMN2* and a c.821C>T variant within exon 6 of the *SMN1* gene. The first plasma sampling for the treatment-naïve SMA subjects was conducted at the age of 10.1 years [IQR: 8.1–11.2]. This cohort included individuals with a chronic phenotype of type 3 disease, which is why they were not prioritized for treatment. Consequently, they lacked motivation to perform a motor evaluation, which resulted in the absence of an assessment of motor status at baseline or before the second sampling.

Type 1 SMA individuals had an average baseline CHOP-INTEND score of 15 ± 7.2, which was significantly lower than the average score of 33.6 ± 7 for type 2 SMA (*p* = 0.0045) and 60.7 ± 5.8 for SMA NBS (*p* = 0.0011). Even though SMA NBS individuals were unlikely to develop type 1 SMA due to an increased *SMN2* copy number, this comparison was undertaken given that CHOP-INTEND was the age-appropriate scale used for the evaluation of these clinically silent SMA children.

There was no significant difference in the baseline HFMSE score between type 2 and type 3 SMA individuals.

### 3.2 Phosphorylated neurofilament heavy chain in cerebrospinal fluid

Type 1 individuals had the highest mean CSF pNF-H level before therapy initiation (2322.1 pg./mL, range 752.8–4810.7 pg./mL). For other groups the mean levels (range) were as follows: 349.7 (105.2–925.4) pg./ml for type 2, 1368.4 (134.9–5767.9) pg./ml for type 3, 1,022 pg./mL for one clinically silent NBS SMA baby who received Nusinersen treatment immediately after being diagnosed at the age of 16 months, and 66.2 (BLQ-194.8) pg./ml for control individuals.

**Figure 1:**
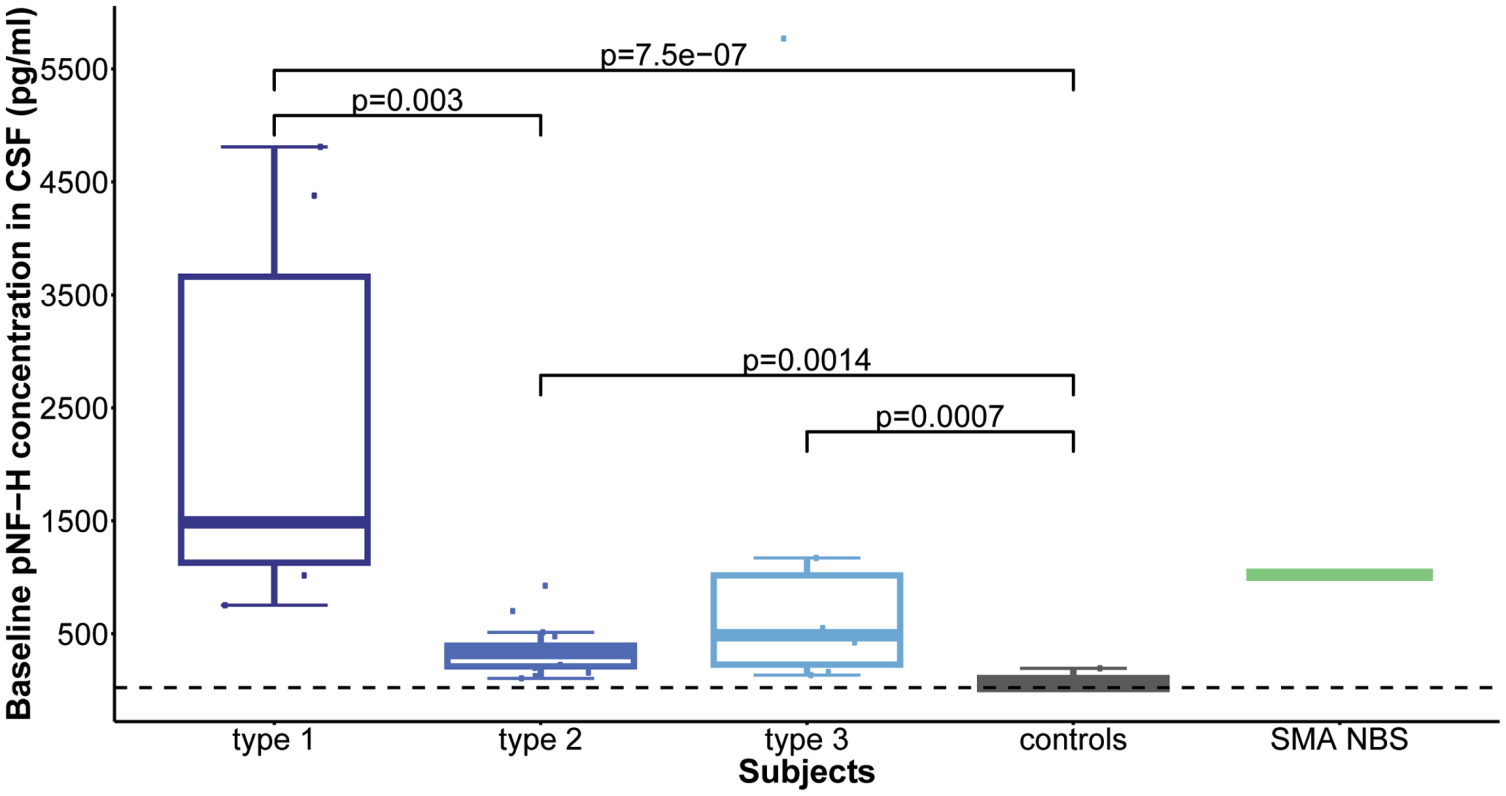
Baseline levels of phosphorylated neurofilament heavy chain (pNF-H) in cerebrospinal fluid (CSF). All individuals with spinal muscular atrophy (SMA) treated with Nusinersen, categorized by type (type 1: *n* = 6; type 2: *n* = 17; type 3: *n* = 6), exhibited markedly elevated mean CSF pNF-H levels compared to controls (*n* = 9). Furthermore, individuals diagnosed with type 1 SMA demonstrated significantly higher mean baseline CSF pNF-H levels than those diagnosed with type 2 SMA.

Statistical analysis revealed that all childhood-onset and Nusinersen treated SMA individuals (type 1, 2, and 3) exhibited significantly elevated mean baseline CSF pNF-H level compared to controls (*p* = 7.5e-07, *p* = 0.0014, *p* = 0.0007, respectively). Comparison of individuals with distinct SMA types showed significant difference in CSF pNF-H levels (*p* = 2.6e-05). Post-hoc analysis unveiled that individuals with type 1 SMA exhibited significantly higher mean baseline CSF pNF-H levels compared to those of type 2 SMA (*p* = 0.003; Figure 1).

Elevated baseline CSF pNF-H level inversely correlated with the following baseline clinical characteristics: age at disease onset (ρ = −0.44, *p* = 0.019), age at first dose (ρ = −0.47, *p* = 0.009), disease duration at baseline (ρ = −0.52, *p* = 0.004) and baseline CHOP-INTEND score (ρ = −0.79, *p* = 0.0008).

**Figure 2:**
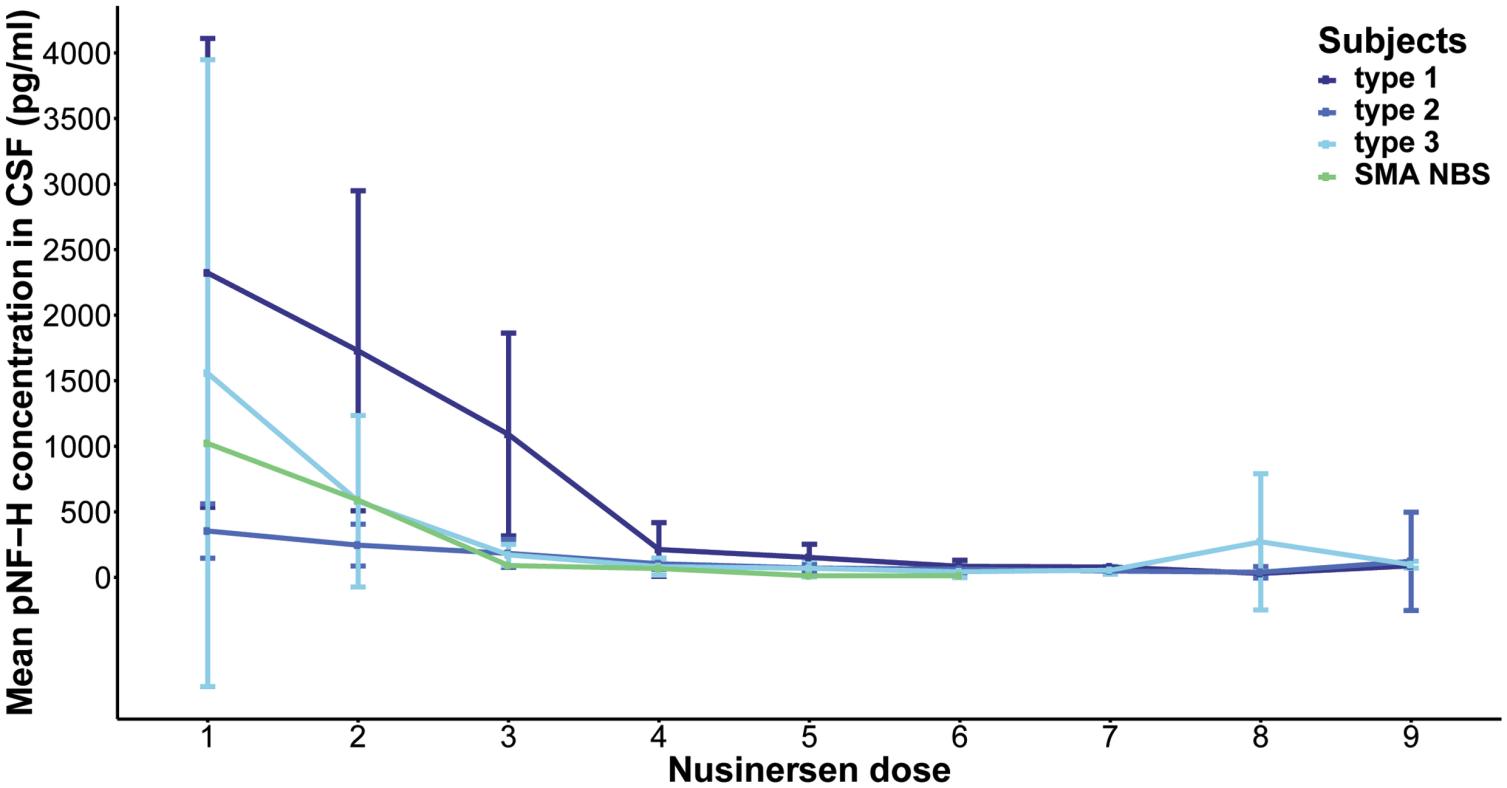
Evolution of phosphorylated neurofilament heavy chain (pNF-H) levels in cerebrospinal fluid (CSF) throughout a 22-month period of Nusinersen treatment in individuals with spinal muscular atrophy (SMA). The initially elevated baseline CSF pNF-H level demonstrated a consistent decrease across all SMA types (type 1, 2, and 3), including one SMA NBS child. This reduction is most pronounced during the first 2 months, coinciding with the loading doses, and subsequently stabilizes at diminished levels throughout the maintenance doses.

Over the course of a 22-month Nusinersen treatment period (before the ninth dose), the initially elevated baseline CSF pNF-H levels decreased across all SMA types, including one SMA NBS child (Figure 2). This reduction was most pronounced during the first 2 months, i.e., during the four loading doses. At the fourth loading dose, the pNF-H concentration reached low levels and remained stable during the subsequent maintenance doses. More precisely, at day 64 there was a 90.8% reduction of the baseline CSF pNF-H level of type 1 individuals, 70.1% in type 2, 93.8% in type 3 and 93.4% in the SMA NBS child. The greatest decline in mean CSF pNF-H level between doses was observed exactly at day 64 in type 1 individuals (877.69 pg./mL), whereas in type 2 and type 3 it was at day 15 (98.66 pg./mL and 857.29 pg./mL, respectively) and at day 29 in the SMA NBS child (499.3 pg./mL).

**Figure 3:**
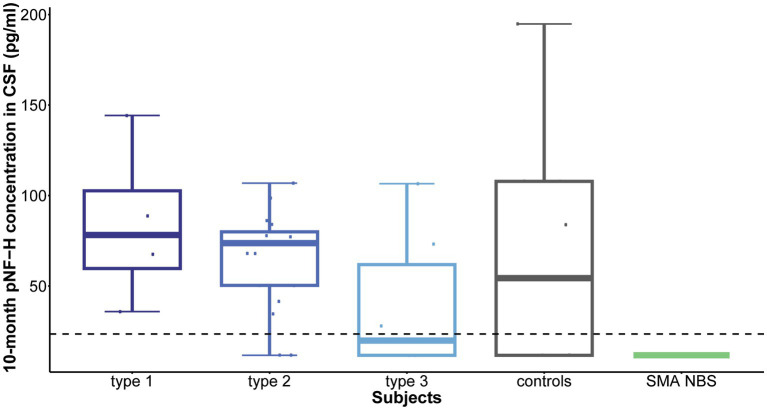
Levels of phosphorylated neurofilament heavy chain (pNF-H) in cerebrospinal fluid (CSF) following a 10-month period of Nusinersen treatment (before the administration of the sixth dose). A notable decrease in CSF pNF-H levels was evident in all individuals with spinal muscular atrophy (SMA) undergoing Nusinersen treatment when compared to their respective baseline levels. Before the sixth dose, the differences in pNF-H levels among SMA types were not statistically significant. Additionally, these pre-sixth dose levels closely resembled those observed in the CSF of control individuals (*n* = 9). Importantly, the baseline pNF-H level for the SMA child identified through newborn screening (SMA NBS) was reduced to below the limit of quantification (<BLQ) before the administration of the sixth dose. The horizontal dashed line in the figure represents the limit of detection of the ELISA assay used (23.5 pg./mL).

**Figure 4:**
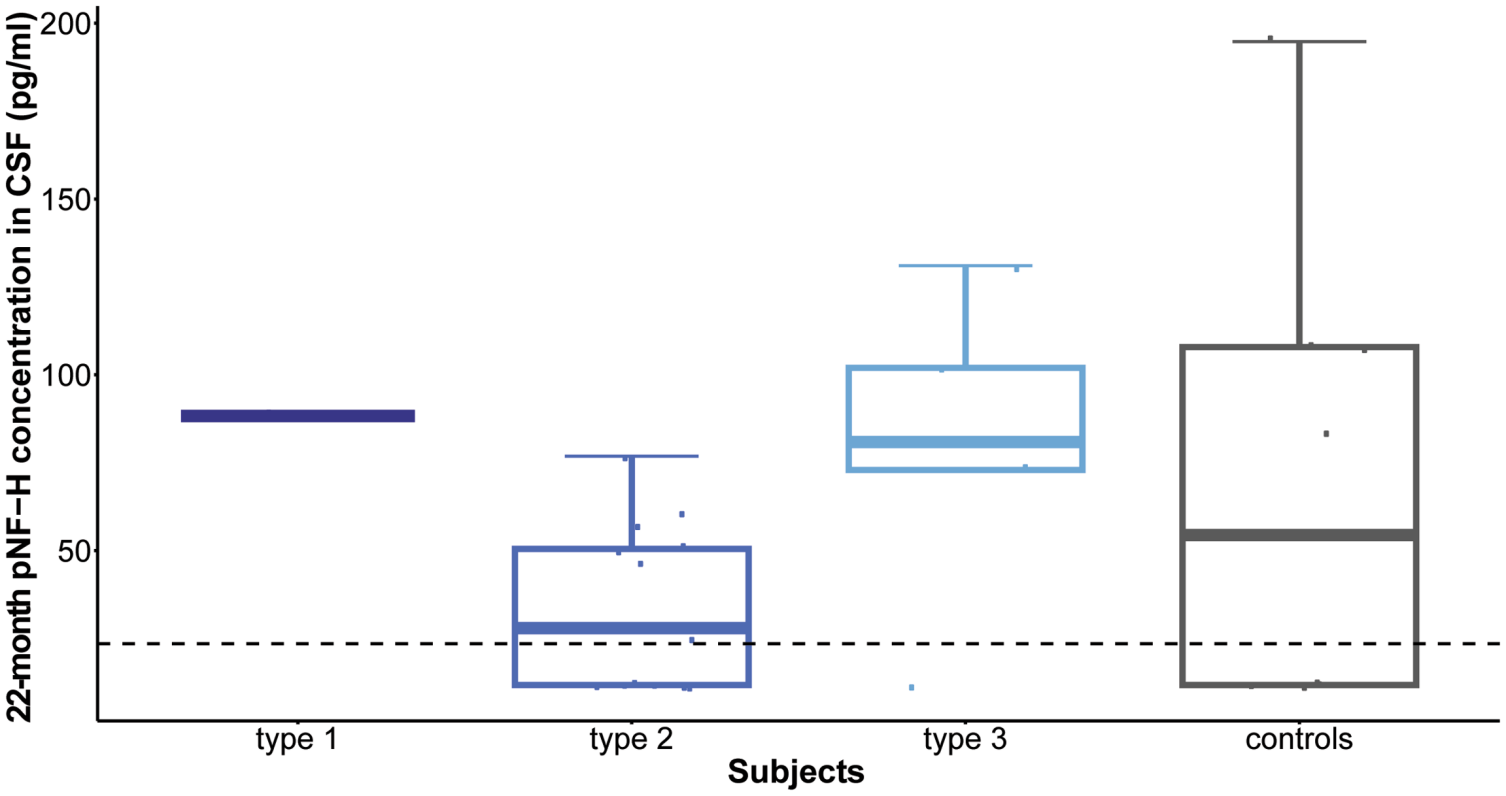
Levels of phosphorylated neurofilament heavy chain (pNF-H) in cerebrospinal fluid (CSF) following a 22-month period of Nusinersen treatment (before the administration of the ninth dose). At the conclusion of the 22-month treatment period, immediately preceding the ninth dose, pNF-H levels remained low and exhibited no statistically significant variation across all three types of spinal muscular atrophy (SMA). Moreover, these levels before the ninth dose closely resembled those observed in the CSF of control individuals (*n* = 9). The horizontal dashed line in the figure represents the limit of detection of the ELISA assay used (23.5 pg./mL).

Following 10 months of treatment (before the sixth dose), a significant reduction was observed in CSF pNF-H levels across all types in comparison to their respective initial levels: *p* = 0.009 for type 1, *p* = 8.4e-07 for type 2, and *p* = 0.005 for type 3. Importantly, CSF pNF-H levels before the sixth dose did not exhibit statistically significant differences between the types (mean CSF pNF-H levels were: 84.1 ± 46 pg./mL for type 1, 64.8 ± 28 pg./mL for type 2, 40.5 ± 40 pg./mL for type 3; *p* = 0.34; Figure 3). Moreover, these pre-sixth dose levels were comparable to those found in the CSF of control individuals (*n* = 9). The baseline level for the NBS SMA child was reduced to <BLQ prior to the administration of the sixth dose. After 22 months, just before the ninth dose, pNF-H levels remained low and displayed no statistically significant difference among all three SMA types (*p* = 0.13; Figure 4). Nonetheless, in comparison to the pre-sixth dose levels, a significant additional reduction in pNF-H levels was observed only in individuals with type 2 (*p* = 0.02).

No association between baseline CSF pNF-H levels and motor function scores after one and 2 years of treatment was observed, nor between changes in motor function and alterations in CSF pNF-H levels over time.

### 3.3 Phosphorylated neurofilament heavy chain in plasma

The aforementioned findings indicate the potential of pNF-H as a biomarker for assessing the response to Nusinersen treatment in CSF, which is accessible during this treatment. However, considering other SMA therapies that do not involve CSF, it was worth investigating whether these trends also apply to plasma.

The average baseline plasma pNF-H level in Nusinersen-treated individuals with type 1 SMA was 4987.08 pg./mL, in type 2 SMA it was 584.96 pg./mL, and in type 3 SMA it was 77.26 pg./mL. Treatment-naïve SMA individuals, control individuals, and individuals with DMD and DM1 showed baseline plasma pNF-H levels < BLQ, as well as asymptomatic siblings identified through newborn screening and possessing 4 *SMN2* copies. In contrast, in one NBS infant with 3 *SMN2* copies, the baseline plasma pNF-H level measured 766.22 pg./mL.

**Figure 5:**
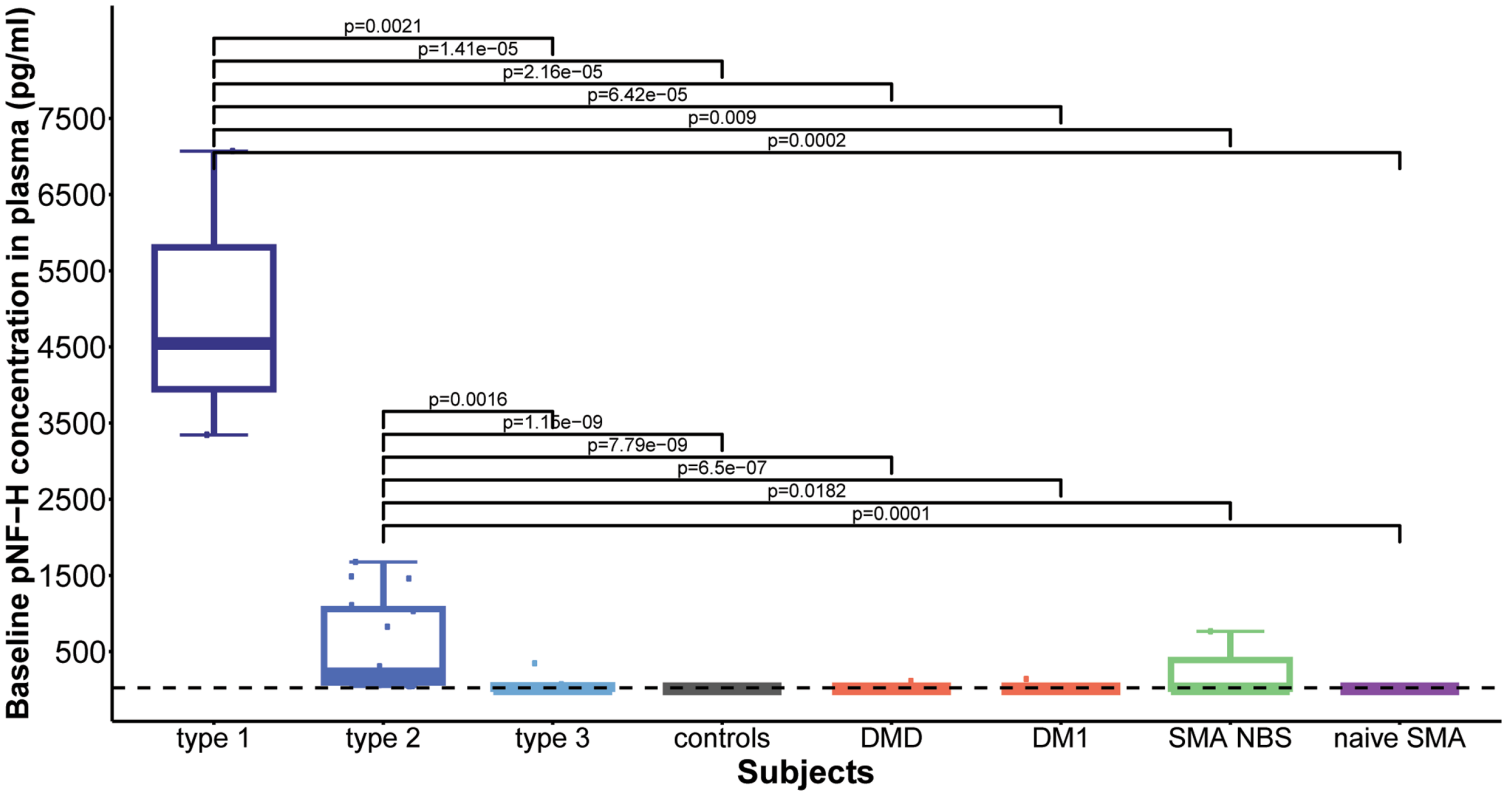
Baseline levels of phosphorylated neurofilament heavy chain (pNF-H) in plasma. Both type 1 and type 2 spinal muscular atrophy (SMA) cohorts exhibited markedly elevated pNF-H levels compared to all other groups, including type 3 SMA individuals, controls, individuals with Duchenne muscular dystrophy (DMD), myotonic dystrophy type 1 (DM1), clinically silent SMA individuals identified via newborn screening (SMA NBS), and untreated SMA individuals with the chronic type 3 form of the disease (naïve SMA). No significant differences were observed in baseline plasma pNF-H levels between SMA types 1 and 2. The horizontal dashed line in the figure represents the limit of detection of the ELISA assay used (23.5 pg./mL).

Baseline plasma pNF-H levels differed significantly among the studied cohorts (*p* = 8.857e-11). Subsequent post-hoc analysis showed that both type 1 and type 2 SMA demonstrated significantly increased plasma pNF-H levels compared to all other groups: type 1 vs. type 3/controls/DMD/ DM1/NBS SMA/naive SMA: *p* = 0.002/*p* = 1.41e-05/*p* = 2.16e-05/*p* = 6.42e-05/*p* = 0.009/*p* = 0.0002; type 2 vs. type 3/ controls/ DMD/DM1/NBS SMA/naïve SMA: *p* = 0.0016/*p* = 1.15e-09/*p* = 7.79e-09/*p* = 6.5e-07/*p* = 0.0182/ *p* = 0.0001 (Figure 5). There was no significant difference in baseline plasma pNF-H levels between types 1 and 2 (*p* = 0.16).

**Figure 6:**
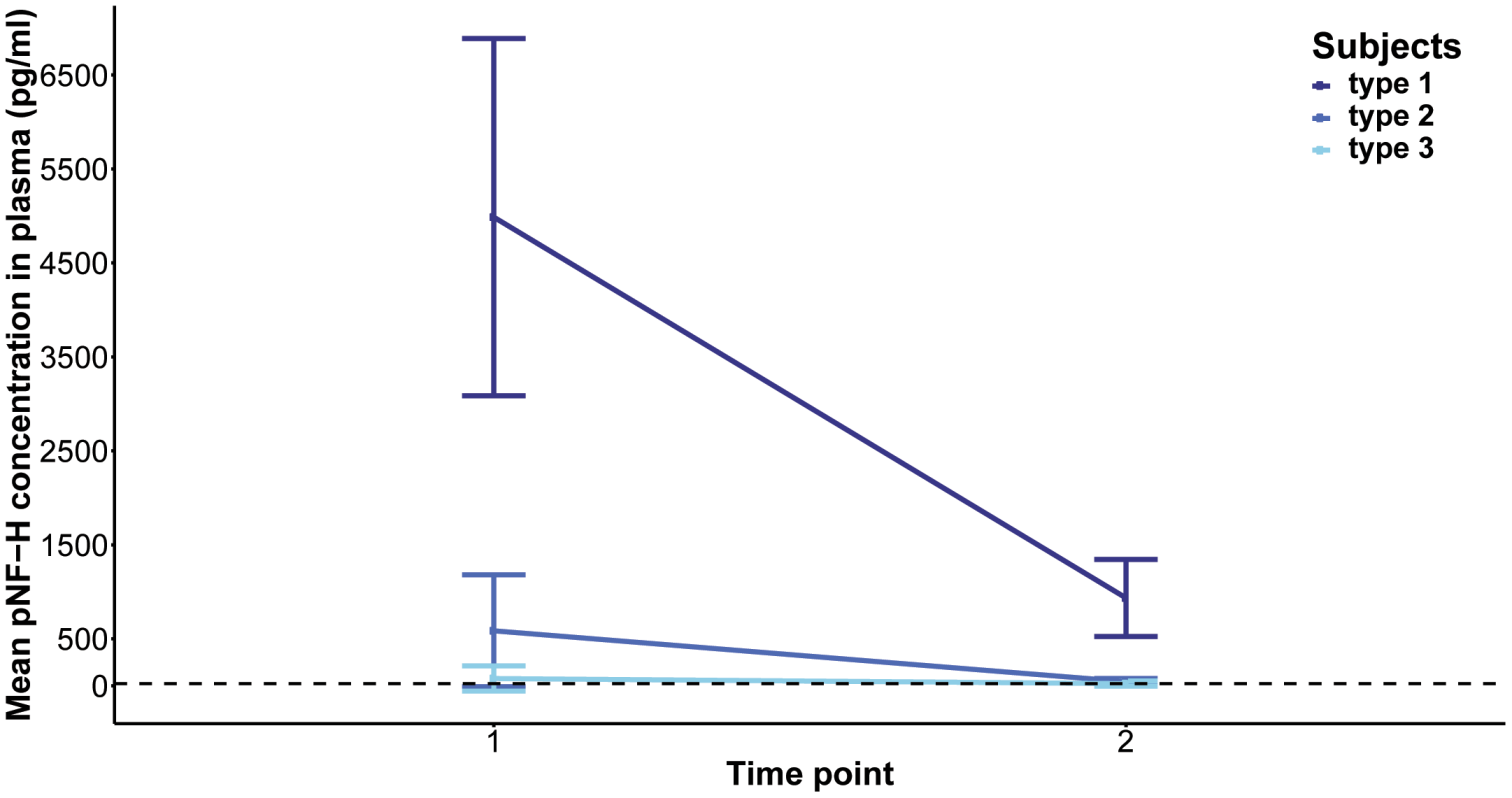
Evolution of phosphorylated neurofilament heavy chain (pNF-H) levels in the plasma of individuals with spinal muscular atrophy (SMA) undergoing Nusinersen treatment. The baseline levels of pNF-H significantly decreased in individuals classified as type 1 SMA after a two-month duration of treatment, and in those categorized as type 2 SMA after 14 months. Conversely, subjects diagnosed with type 3 SMA, characterized by lower initial pNF-H levels, did not exhibit significant alterations in plasma pNF-H concentrations following the 14-month treatment period.

Elevated baseline plasma pNF-H levels exhibited a significant reduction in type 1 following 2 months of treatment (*p* = 0.06, mean level = 935 pg./mL) and in type 2 after 14 months (*p* = 5.407e-05, mean level = 44.46 pg./mL). In contrast, individuals with type 3 SMA, characterized by lower baseline pNF-H levels, demonstrated no significant fluctuations in plasma pNF-H levels after 14 months (*p* = 0.6, mean level = 23.27 pg./mL; Figure 6). In treatment-naïve SMA individuals, the pNF-H level remained <BLQ after 3 years.

Levels of pNF-H in CSF and plasma of SMA individuals treated with Nuisnersen are presented in the Supplementary Table 1.

## 4 Discussion

This study compares plasma and CSF pNF-H levels among Nusinersen-treated individuals with symptomatic SMA across childhood-onset types. To our knowledge, this study represents the only one to compare these pNF-H levels with those of presymptomatic SMA individuals, treatment-naïve individuals with chronic SMA type 3, cohorts of controls and individuals with neuromuscular disorders unrelated to motor neuron degeneration. Therefore, we believe that our study has several advantages. Primarily, it conducted comparisons across various cohorts employing a uniform methodology. This approach facilitates comparisons, as divergent methodologies may yield disparate pNF-H levels, thereby complicating inter-study comparisons. In addition, this study has the advantage of assessing CSF samples from controls, in addition to plasma samples. Furthermore, this study includes samples of clinically silent infants with SMA, a cohort expected to increase in future due to the implementation of mandatory newborn screening for SMA in Serbia in September 2023.

Our findings suggested that CSF pNF-H levels may differentiate SMA individuals from controls and serve as an indicator of neurodegeneration across all childhood-onset types of SMA, as evidenced by elevated levels observed in all SMA individuals in comparison to controls. In contrast to these observations, existing literature reports low baseline CSF pNF-H levels in type 2 and type 3 SMA, without significant differences from controls (14–17). Moreover, our results suggest that CSF pNF-H levels may reflect disease severity, as individuals with SMA type 1 had the highest average CSF pNF-H levels before initiation of therapy. These findings on type 1 SMA align with the study of Johannsen et al. (12), one of the limited inquiries examining CSF pNF-H levels among this specific SMA subgroup, similarly highlighting higher initial pNF-H values in type 1 SMA individuals compared to types 2 and 3. However, these results should be interpreted with caution, as there is no specific threshold value of pNF-H in CSF that would allow the definitive anticipation of the clinical manifestation of the disease.

In our study, we observed an inverse correlation between elevated baseline CSF pNF-H levels and more severe baseline clinical characteristics, consistent with prior investigations (11, 12). Although improvements in motor function were observed among our Nusinersen-treated cohorts, we did not observe a significant correlation between baseline CSF pNF-H levels and future motor outcomes, nor between changes in CSF pNF-H levels over time and changes in motor function. Although the degree of motor neuron degeneration can significantly influence clinical outcomes, the relationship between baseline CSF pNF-H levels and assessed motor function is complex. Thus, pretreatment pNF-H CSF levels, at least in our study, have no predictive value. This holds particular significance for presymptomatic individuals identified through newborn screening programs.

In the current study, plasma pNF-levels reflect increased neuronal damage before treatment in both type 1 and type 2 SMA individuals, who exhibited higher baseline pNF-H levels compared to type 3, controls, DMD and DM1 groups, as well as compared to SMA NBS children and treatment-naïve SMA individuals. Existing literature consistently reports high plasma pNF-H levels in type 1 SMA individuals, significantly surpassing those of healthy controls (11), whereas adolescent and adult SMA individuals did not exhibit elevated plasma pNF-H levels compared to controls, neither initially nor later during the treatment (13–17). This could suggest that plasma pNF-H levels may not be a predictive biomarker for disease progression and/or treatment response in adults with SMA due to depletion of the motor pool resulting from chronic disease (18), in contrast to infants with SMA type 1, where the course of the disease progresses faster with more acute neuronal damage. Another possibility, as suggested, is that Nusinersen treatment in adults leads to an overall stabilization, as opposed to the more pronounced improvements observed in infants (13). Our comparison of plasma pNF-H levels between SMA individuals and individuals with DMD and DM1 revealed that heightened pNF-H levels were not observed in subjects with inherited non-motor neuron diseases. This indicates that, although elevated levels of pNF-H are not only associated with SMA, they nevertheless specifically reflect the pathology of this disease. Furthermore, our findings on CSF and plasma pNF-H levels in clinically silent infants with SMA suggest that even at a very early stage, they may be indicative of the presence of neuronal degeneration that may not be apparent through motor scale assessment. These results are in line with findings of the NURTURE clinical trial (19) and emphasize the limited utility of motor scale assessments, particularly in newborns, the capacity of pNF-H to delineate presymptomatic phases of the disease (20), and the potential of pNF-H in plasma to influence decisions regarding the initiation of therapy in presymptomatic individuals identified through newborn screening. In light of these findings, we decided to routinely assess pNF-H levels in all infants identified by newborn screening for SMA in our country.

Furthermore, neurofilaments emerged as valuable biomarkers for monitoring treatment response. Given their status as markers of neuronal injury, it is anticipated that neuroprotective interventions would result in diminished neurofilament levels. Our findings distinctly reveal that throughout the course of Nusinersen treatment, CSF pNF-H level exhibits a decline during the loading doses, followed by stabilization at a reduced level starting from the initial maintenance dose. This diminished level persists throughout the follow-up period, leading to statistically significant reductions after 10 and 22 months. The ultimate levels exhibit a remarkable uniformity across all patient cohorts, showing no statistically significant differences between SMA types or when compared to controls. Therefore, our findings suggest that monitoring CSF pNF-H levels could function as a standardized metric to assess rapid short-term response to treatment in individuals with SMA, regardless of disease subtype. As the low level of pNF-H in CSF persists even 22 months after initiation of therapy, pNF-H could further serve as an indicator of long-term suppression of neurodegeneration. Regarding the dynamics of plasma pNF-H levels, a statistically significant decrease was observed in type 1 and type 2 during treatment with Nusinersen at the second time point, occurring after 2 months for type 1 and 14 months for type 2. However, there was no significant difference in plasma pNF-H levels observed in type 3 compared to a later time period of 14 months (second time point). Similar discrepancy in type 3 pNF-H levels between CSF and plasma was previously documented (13, 14), showing that CSF pNF-H levels exhibited a significant decline during treatment with Nusinersen, while pNF-H concentrations in serum demonstrated no changes from baseline.

Our study has several limitations. First, it addresses motor neuron degeneration, which is only one aspect of SMA pathophysiology, and examines a single protein biomarker across all cohorts. In addition, the sample size is relatively modest, and further decreases during the follow-up period. Accordingly, careful interpretation of all findings is necessary.

In conclusion, our findings, combined with existing literature data, contribute to a growing body of evidence suggesting that in response to Nusinersen treatment, pNF-H levels vary among different types of SMA, as well as between CSF and plasma/serum. In particular, our observations indicate that CSF pNF-H levels are an informative biomarker for disease onset and exhibit promise for assessing the efficacy of Nusinersen therapy. Initiation of treatment with Nusinersen typically results in a rapid decrease of CSF pNF-H levels followed by stabilization, approaching levels observed in controls. However, plasma pNF-H levels show limited utility in detecting motor neuron degeneration, assessing its suppression, and reflecting therapeutic effects, particularly in individuals with SMA type 3 and a chronic disease phenotype. These findings underscore the need for further investigation of the applicability of pNF-H as a standalone candidate biomarker (21), including SMA individuals undergoing alternative SMN-dependent therapies, particularly within chronic SMA subgroups. Considering the rarity of the disease, the high cost of available therapies, as well as the projected decrease in symptomatic SMA cases in the future, a multicenter approach is recommended.

## Data availability statement

The raw data supporting the conclusions of this article will be made available by the authors, without undue reservation.

## Ethics statement

The studies involving humans were approved by Clinic for Neurology and Psychiatry for Children and Youth, Belgrade; University Children’s Hospital Tirsova, University Clinical Centre of Serbia, Belgrade; and Neurology Clinic, University Clinical Centre of Serbia, Belgrade. The studies were conducted in accordance with the local legislation and institutional requirements. Written informed consent for participation in this study was provided by the participants’ legal guardians/next of kin.

## Author contributions

MB: Conceptualization, Investigation, Writing – original draft. AK: Resources, Writing – review & editing. VB-S: Resources, Writing – review & editing. KJ: Resources, Writing – review & editing. SP: Resources, Writing – review & editing. JK: Formal analysis, Writing – review & editing. SMJ: Investigation, Writing – review & editing. NG: Investigation, Visualization, Writing – review & editing. JP: Formal analysis, Writing – review & editing. DN: Resources, Writing – review & editing. ZS: Resources, Writing – review & editing. GB: Writing – review & editing. VM-R: Writing – review & editing, Resources. DS-P: Supervision, Writing – review & editing.
